# Patient-level and program-level monitoring and evaluation of differentiated service delivery for HIV: a pragmatic and parsimonious approach is needed

**DOI:** 10.1097/QAD.0000000000001723

**Published:** 2018-01-11

**Authors:** William J. Reidy, Miriam Rabkin, Maureen Syowai, Andrea Schaaf, Wafaa M. El-Sadr

**Affiliations:** a ICAP at Columbia University; b Department of Epidemiology, Mailman School of Public Health, Columbia University, New York, New York, USA.

**Keywords:** antiretroviral, differentiated care, differentiated service delivery, evaluation, global, HIV, implementation, monitoring

## Background

Achieving the ambitious global HIV treatment goals will require a marked expansion of antiretroviral therapy (ART) coverage and close attention to HIV service quality [1]. In response, a growing body of evidence supports the use of differentiated ART services (DARTS) for subgroups of HIV-positive individuals [2–14]. Although differentiated services can support diverse patient groups, most current DARTS initiatives target stable adult patients, that is, those demonstrating ART adherence and viral suppression or viral suppression alone [15]. Both community-based and facility-based DARTS can reduce the burdens associated with frequent and lengthy clinic visits for both patients and health providers. Ultimately, these models aim to enhance retention, ART adherence, viral suppression, and quality of life [16].

The implementation of such novel approaches involves fundamental changes to delivery systems. This can make monitoring services more challenging, requiring new approaches to ensure that data are available to inform the care of individuals engaged in DARTS and enable programmatic evaluation of diverse DARTS models.

Important lessons have been learned regarding HIV program monitoring and evaluation (M&E). These include the critical importance of parsimony and pragmatism, focusing on information that is feasible to collect and essential for patient and program management [17]. These principles are particularly important as existing M&E systems evolve in response to increasingly diverse DARTS models.

This article highlights common gaps in M&E systems with respect to DARTS. We outline elements of an integrated, streamlined approach that provides robust information to guide patient and program management, while minimizing the burden of data collection and aggregation. Lastly, we note that additional information is needed for assessment and strategic planning at the health system level; this may require additional data collection and/or special studies.

## Patient-level data: documenting eligibility for and engagement in differentiated antiretroviral therapy services

An increasing number of countries endorse the use of DARTS, such as facility-based adherence clubs, community-based antiretroviral groups, fast-track visits, outreach services, and/or community drug pickups [18]. In order to take DARTS to scale, it is critically important that providers are able to identify which patients are eligible for DARTS, given current local guidelines; whether they are enrolled in DARTS; and in which model they are participating. As eligibility may change over time, this information will need to be updated at each visit. Additionally, providers need to know at a glance whether and when patients have received their ART medication and required laboratory assessments, as well as their adherence levels and psychosocial support needs.

At present, standardized, structured approaches to document this information are scarce. Existing tools were not designed to capture patient-level information relevant to DARTS and, thus, are limited in guiding providers through the key steps in a DARTS patient visit. Modification of the ART medical record form will be required to capture these unique elements. Using the ART medical record, which is ubiquitous across countries, as the primary repository of DARTS information is essential to avoid the fragmentation that occurs whenever some information is documented at the clinic and some only in separate forms to be completed in the community, the pharmacy, or the laboratory.

In addition to a modified ART record, supplementary tools are needed to collect information regarding membership in patient DARTS groups [19], and to capture essential information from encounters that occur outside the facility (e.g. community ART groups), such as new symptoms, especially those that might indicate tuberculosis, adherence with ART and pregnancy status, as well as other information that may necessitate timely follow-up and referral for appropriate complementary services. These data should be regularly communicated with staff at the health facility wherever the patient receives clinical care, to trigger appropriate action such as recall to the health facility; they should also be transcribed into each patient's ART record.

## Program-level data: understanding differentiated antiretroviral therapy services coverage and performance

**Fig. 1:**
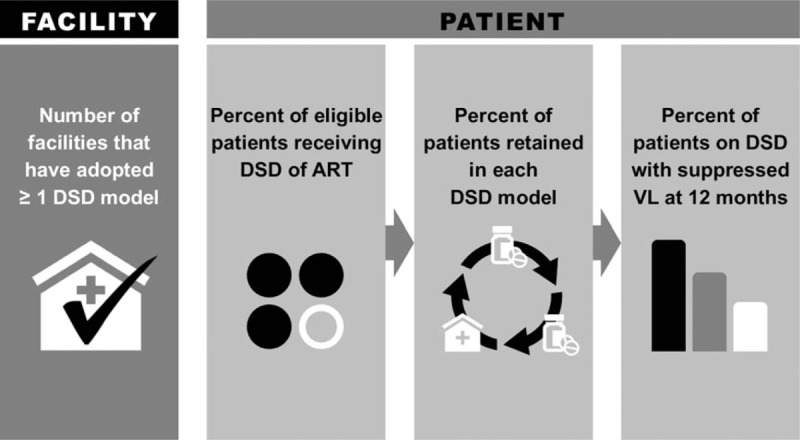
Indicators for differentiated antiretroviral therapy.

At the program level, information for monitoring and evaluating DARTS models is also important, particularly early in their implementation and scale-up. Examples of relevant programmatic questions include the proportion of health facilities offering DARTS, the proportion of eligible patients enrolled in DARTS, and the proportion of patients enrolled in specific DARTS models who are retained and achieved durable viral suppression (Fig. 1). Some of this information may also be useful at district, provincial, or regional levels, for example, to compare coverage of DARTS and patient outcomes within and between localities; (though, cross-country comparisons may be limited by varying eligibility criteria for DARTS).

Unfortunately, existing widely used ART M&E systems are not yet able to measure the performance of DARTS in this way [20,21]. To describe DARTS coverage, uptake, and outcomes, M&E systems will need to collect and summarize the types of data described above. Depending on local resources and priorities, such data may be disaggregated by type of DARTS model, age, sex, and key population group.

In some contexts, tracking and manually calculating a new set of indicators using paper registers may not be feasible or advisable. However, countries with patient-level electronic ART databases may add data elements to capture DARTS eligibility and engagement for every patient at every visit. In such scenarios, it is feasible to aggregate patient data in an automated fashion and easily describe DARTS model coverage, and outcomes.

## Health system-level

Some questions regarding the impact of DARTS at the health system level (e.g. patient/provider satisfaction, wait times, efficiency, and cost) can best be addressed via periodic data collection and/or special studies. Country programs may choose to convene annual/semiannual data review meetings, wherein facility-level or district-level staff present detailed information on a sample of patient records, as a complement to routinely reported information. Data collection and discussions could be focused on areas of high interest, such as specific quality improvement interventions. This could be supplemented with periodic assessments of provider–patient load, wait times, satisfaction, and measures to inform cost-effectiveness calculations.

## Conclusion

As differentiated ART is implemented broadly, country programs, funders, and program managers will seek information on the coverage and performance of such models. Refinement of existing M&E systems is, therefore, required. Efforts to ‘differentiate’ M&E systems must be parsimonious and pragmatic; enabling data-driven learning to optimize services and outcomes.

## Acknowledgements

The authors wish to acknowledge the contributions by the following individuals: Peter Ehrenkranz, Tiffany Harris, Maria Lahuerta, Ruby Fayorsey, and Fatima Tsiouris.

Funding: The authors acknowledge funding support from the Bill and Melinda Gates Foundation.

Authors’ contributions: W.R., M.R., M.S., and W.E.S. conceptualized the article. W.R., M.R., and A.S. wrote the initial draft and A.S. developed Figure 1. All authors reviewed and approved the final draft and approved of the decision to submit the manuscript for publication.

### Conflicts of interest

There are no conflicts of interest.
